# Shifting of Physicochemical and Biological Characteristics of Coffee Roasting Under Ultrasound-Assisted Extraction

**DOI:** 10.3389/fnut.2021.724591

**Published:** 2021-08-20

**Authors:** Acharaporn Duangjai, Surasak Saokaew, Bey-Hing Goh, Pochamana Phisalprapa

**Affiliations:** ^1^Unit of Excellence in Research and Product Development of Coffee, Division of Physiology, School of Medical Sciences, University of Phayao, Phayao, Thailand; ^2^School of Pharmaceutical Sciences, Center of Health Outcomes Research and Therapeutic Safety (Cohorts), University of Phayao, Phayao, Thailand; ^3^Unit of Excellence on Clinical Outcomes Research and IntegratioN (UNICORN), School of Pharmaceutical Sciences, University of Phayao, Phayao, Thailand; ^4^Unit of Excellence on Herbal Medicine, School of Pharmaceutical Sciences, University of Phayao, Phayao, Thailand; ^5^Division of Pharmacy Practice, Department of Pharmaceutical Care, School of Pharmaceutical Sciences, University of Phayao, Phayao, Thailand; ^6^Biofunctional Molecule Exploratory Research Group (BMEX), School of Pharmacy, Monash University Malaysia, Subang Jaya, Malaysia; ^7^College of Pharmaceutical Sciences, Zhejiang University, Hangzhou, China; ^8^Division of Ambulatory Medicine, Department of Medicine, Faculty of Medicine Siriraj Hospital, Mahidol University, Bangkok, Thailand

**Keywords:** coffee roasting, arabica, ultrasound-assisted extraction, chlorogenic acid, antioxidant

## Abstract

Ultrasound-assisted extraction (UAE) is an effective tool for the extraction of natural antioxidants. Thus, differentially roasted Arabica-coffee beans known as light (LC), medium (MC), and dark coffee (DC) were prepared and extracted under the influence of UAE. Following that, they were examined specifically on theirs physicochemical and biological characteristics: nutritional values, pH, °Brix, antioxidant activities, polyphenol content, caffeine, and chlorogenic-acid levels. Various parameters, such as extraction temperatures (20, 40, and 80°C) and extraction time periods (5, 10, and 20 min), were examined. DC extract was less acidic than those on MC and LC extracts. LC showed higher moisture content than the MC and DC (1.56, 1.3, and 0.92%, respectively). MC displayed the highest polyphenol content and potent antioxidant activity. Caffeine and chlorogenic acid contents trend to decrease during roasting. The maximum caffeine level was found in MC at 80°C for 5 min (27.65 mg/g extract). The highest chlorogenic acid content was in LC at 80°C for 10 min (16.67 mg/g extract). The caffeine and chlorogenic acid contents were related to the polyphenol content and depended on the roasting and extraction conditions. These results suggest that the UAE at various temperature and extraction time period may alter the physicochemical and biological characteristics of different coffee roasts.

## Introduction

A drink or beverage is deemed as a specific liquid or concoction intended for human consumption. They are generally divided into two main categories which are known as alcoholic and non-alcoholic beverages. Apart from satisfying the thirst, drinks are known to play critical roles in human culture such as Cha-no-yu, the very famous Japanese tea ceremony (1) and the uniquely Ethiopian coffee ceremony known as “Buna” (2). Each of them carries a very deep specific meaning and representative of their very own specific ethnic group and cultural identity.

Among various types of beverages commonly being consumed, coffee is deemed as one of the most popular drinks in the world. Statistically, the worldwide consumption of coffee in 2020 had reached approximately 10 million tons (3). The one of kind characteristic flavor, taste, and lavishness of coffee aroma might have appeared as key reasons on why coffee beverages are so adored by customers. With the growing health consciousness among the world population, there is more consideration than ever on the health and wellness benefits of nourishment or drink that would bring to humans.

Coffee belongs to the Rubiaceae family and genus of *Coffea*. Coffee consumption is connected to numerous health benefits, such as improved brain function (4), anti-inflammatory activity (5), reduced risk of type 2 diabetes (6), and protection against liver disease (7). Coffee beans contain a variety of compounds with powerful antioxidant activity, including caffeine, chlorogenic acid, diterpenes, and trigonelline (8). Nonetheless, the bioactive compound composition in coffee beans depends on several factors, including the bean variety, place of origin, growing conditions (9, 10), and especially the roasting process and extraction temperature. During roasting, coffee beans undergo changes in their physical and chemical composition that are controlled by the roasting time and temperature. The degree of roasting can be monitored by comparing the color of the roasted beans as a light, medium, or dark (11). Different coffee roasting parameters are known to impose an appearance change on the antioxidant activity and polyphenol content (12). Furthermore, coffee brewing refers to the extraction process, including the extraction pressure, coffee-to-water ratio, water quality, contact time, particle size distribution, and temperature (13). These factors lead to distinct chemical and biological properties (10). Therefore, it is deemed critical to determine these optimum parameters to be able to produce a product that is retaining its biofunctionalities and at the same time retaining the unique delicacy of cuppa coffee.

Conventional extraction is a common method used to obtain phenolic compounds. However, the process leads to the loss of phenolic compounds due to high temperature and long extraction time periods (14). Ultrasound-assisted extraction (UAE) is an efficient method for the extraction of natural antioxidants from plant materials (15) because it reduces the time required for extraction and energy expenditure and enhances the yield of the bioactive compounds (16). In view of the great advantages possessed by UAE, the current study has been designed in a way to examine the pH, °Brix, antioxidant activities, polyphenol content, and phenolic compounds following the integration of UAE during the coffee roasting process.

## Materials and Methods

### Chemicals

The Folin–Ciocalteu reagent, 2,2′-diphenyl-1-picrylhydrazyl (DPPH), 2,2′-azino-bis(3-ethylbenzothiazoline-6-sulfonic acid) (ABTS), and gallic acid were purchased from Sigma-Aldrich Co. (St. Louis, MO, USA). All other chemical reagents used in this study were of analytical grade.

### Plant Materials

Green coffee beans (*Coffea arabica*) were obtained from the Chao-Thai-Pukao Factory, Chiang Mai, Thailand. A voucher specimen of the coffee tree was deposited in the PNU herbarium of the Faculty of Biology, Naresuan University, Phitsanulok, Thailand, under collection number NU003806. Green coffee beans were roasted at distinct temperatures and time periods to obtain light, medium, and dark roasts under 10 to 20 min and 350 to 450°F (176.7-232.2°C). The degree of roasting was determined by the bean color change to brown, dark brown, and black, respectively. Roasted coffee beans (light, medium, and dark) were subjected to extraction with water (1:5 w/v sample to water) using an ultrasonic bath operating at 35 kHz at 20, 40, or 80°C for 5, 10, or 20 min. After sonication, the solution was filtered and freeze-dried (ScanVac CoolSafe 110-4 Pro, Electronex, India). The crude light coffee (LC), medium coffee (MC), and dark coffee (DC) extracts were stored at −20°C until further analysis.

### Analytical and Proximate Analyses

The pH of coffee roast extracts was measured with a pH meter. Sugar levels of the extracts were determined as °Brix using a digital refractometer. The moisture, ash, protein, fat, and fiber contents were determined according to the methods described by the Association of Official Analytical Chemist (AOAC) International (17).

### High-Performance Liquid Chromatography Analysis

Coffee roast extracts were subjected to high-performance liquid chromatography (HPLC) to determine the levels of caffeine and chlorogenic acid. The HPLC separation was performed on a C18 column using mobile phase A (15% methanol) and mobile phase B (85% methanol:distilled water [30:70], 2% acetic acid; pH 3.4), at a flow rate of 0.5 ml/min with detection at 320 nm for chlorogenic acid and at 280 nm for caffeine. The peaks were identified by the reference standards.

### Determination of Total Polyphenols

The total polyphenol content of coffee roast extracts was determined using the Folin–Ciocalteu method (18). Gallic acid was used as a standard. Each coffee roast extract (1 mg/ml) was mixed with the Folin–Ciocalteu reagent; sodium carbonate (7.5%) was added to the mixture, and it was incubated for 30 min. The reaction was measured at 750 nm. The total polyphenol content is expressed as gallic acid equivalents (GAE) in milligrams per gram extract.

### Antioxidant Activity Determination by DPPH Radical Scavenging Assay

The antioxidant activity of coffee roast extracts was measured using the DPPH assay, as previously described (19). The extracts were mixed with methanolic DPPH solution and incubated in the dark for 30 min. Absorbance was measured at 510 nm. Antioxidant activity is expressed as 50% of the radical scavenging activity (IC_50_).

### Antioxidant Activity Determination by ABTS Radical Scavenging Assay

The antioxidant activity of coffee roast extracts was measured by the ABTS radical cation decolorization assay (20) with minor modifications. The ABTS cation radical reagent was produced by reacting ABTS solution with potassium persulfate solution in the dark for 12–16 h. Each coffee roast extract (100 μl) was incubated with the ABTS reagent (100 μl) at room temperature for 30 min. Then, the absorbance was measured at 734 nm. The results were expressed as the IC50.

### Antioxidant Activity Determination by FRAP Assay

The FRAP was estimated according to the previously reported method (21). The method is based on the reduction of the Fe^3+^-tripyridyl triazine (TPTZ) to Fe^2+^-TPTZ. The FRAP reagent was prepared by mixing acetate buffer solution, TPTZ, and FeCl_3_·6H_2_O. Each coffee roast extract (50 μl) was incubated with the FRAP reagent (150 μl) at 37°C for 30 min. The absorbance was read at 593 nm. All the determinations were performed in triplicates. The results were expressed as μM Fe^2+^ per gram extract.

### Statistical Analyses

Results were presented as the mean ± SEM. Data was evaluated using one-way ANOVA as appropriate by utilizing the StatPlus data analysis package in Microsoft Excel Version 15 (Microsoft Corp., Redmond, WA, USA). The *p*-values below 0.05 were considered statistically significant.

## Results

The nutritional composition of coffee roast extracts was evaluated by determining their moisture, fiber, ash, fat, protein, nitrogen-free extract (NFE), and energy content (Table 1). Coffee beans were roasted for 10, 15, and 20 min and 350, 400, and 450°F, respectively, to obtain LC, MC, and DC. The moisture content of LC (1.56%) was higher than MC (1.3%) and DC (0.92%). Furthermore, the coffee roast extracts contained 98.44–99.08% dry matter, 16.20–17.93% fiber, 4.28–4.52% ash, 14.04–15.83% fat, 15.21–16.23% protein, 46.41–47.78% NFE, and 375.53–393.65 kcal energy per 100 g on a dry weight basis.

**Table 1:** Nutritional values of the coffee roast extracts.

	**Roasting temperature (^**°**^F)**	**%Moisture**	**%Dry matter**	**%Fiber**	**%Ash**	**%Fat**	**%Protein**	**%NFE**	**Energy/100 g (Kcal)**
Light coffee (LC)	350	1.6 ± 0.03	98.4 ± 0.03	18 ± 0.6	4.3 ± 0.1	14.04 ± 1	15.2 ± 0.2	47 ± 1	367 ± 5
Medium coffee (MC)	400	1.3 ± 0.1	99 ± 0.1	17 ± 0.5	4.5 ± 0.1	14.2 ± 0.1	15.3 ± 0.2	48 ± 0.3	380.2 ± 1.2
Dark coffee (DC)	450	0.92 ± 0.03	99.1 ± 0.03	16.2 ± 0.6	4.3 ± 0.03	16 ± 0.4	16.2 ± 0.2	46 ± 1	393.7 ± 4
*P*-value^*^		<0.001	<0.001	0.026	0.036	0.003	<0.001	0.036	<0.001

*Data are expressed as mean ± SEM*,

* *p-values were tested using the ANOVA test, when statistically significant (p < 0.05) post-hoc were tested using the Bonferroni test and found that dark coffee (DC) was significantly different from those to the others*.

*NFE, nitrogen free extract*.

It is possible that the different UAE temperatures and time periods affect the acidity and total soluble solids (°Brix) of the coffee roast extracts. Indeed, the DC extract was less acidic than the MC and LC extracts. However, the pH varied depending on the extraction temperature (20, 40, or 80°C) and time (5, 10, or 20 min). In addition, any changes in the total soluble solid content of the coffee roast extracts are listed in Table 2.

**Table 2:** pH and °Brix of coffee roast extracts according to extraction temperature and time.

	**Extraction temperature (^**°**^C)**	**pH**			^****°****^ **Brix**		
		**5 min**	**10 min**	**20 min**	**5 min**	**10 min**	**20 min**
Light coffee (LC)	20	5.1 ± 0.01	5.1 ± 0.02	5.1 ± 0.01	2 ± 0	2 ± 0	1.6 ± 0.4
	40	5.5 ± 0.01	5.1 ± 0.01	5.1 ± 0.00	1 ± 0	2 ± 0	1.8 ± 0.3
	80	5.1 ± 0.02	5.1 ± 0.01	5. ± 0.02	2 ± 0	2 ± 0	2 ± 0
Medium coffee (MC)	20	5.1 ± 0.03	5.1 ± 0.02	5.5 ± 0.02	2 ± 0	2 ± 0	2 ± 0
	40	5.5 ± 0.01	5.5 ± 0.01	5.1 ± 0.01	1.7 ± 0.6	2 ± 0	2 ± 0
	80	5.5 ± 0.02	5.5 ± 0.01	5.4 ± 0.01	2 ± 0	2 ± 0	2 ± 0
Dark coffee (DC)	20	5.8 ± 0.1	5.7 ± 0.02	5.6 ± 0.01	2 ± 0	2 ± 0	2 ± 0
	40	5.8 ± 0.03	5.7 ± 0.1	5.5 ± 0.01	2 ± 0	2 ± 0	2 ± 0
	80	5.6 ± 0.01	5.6 ± 0.01	5.4 ± 0.01	2 ± 0	2 ± 0	2 ± 0

**Figure 1:**
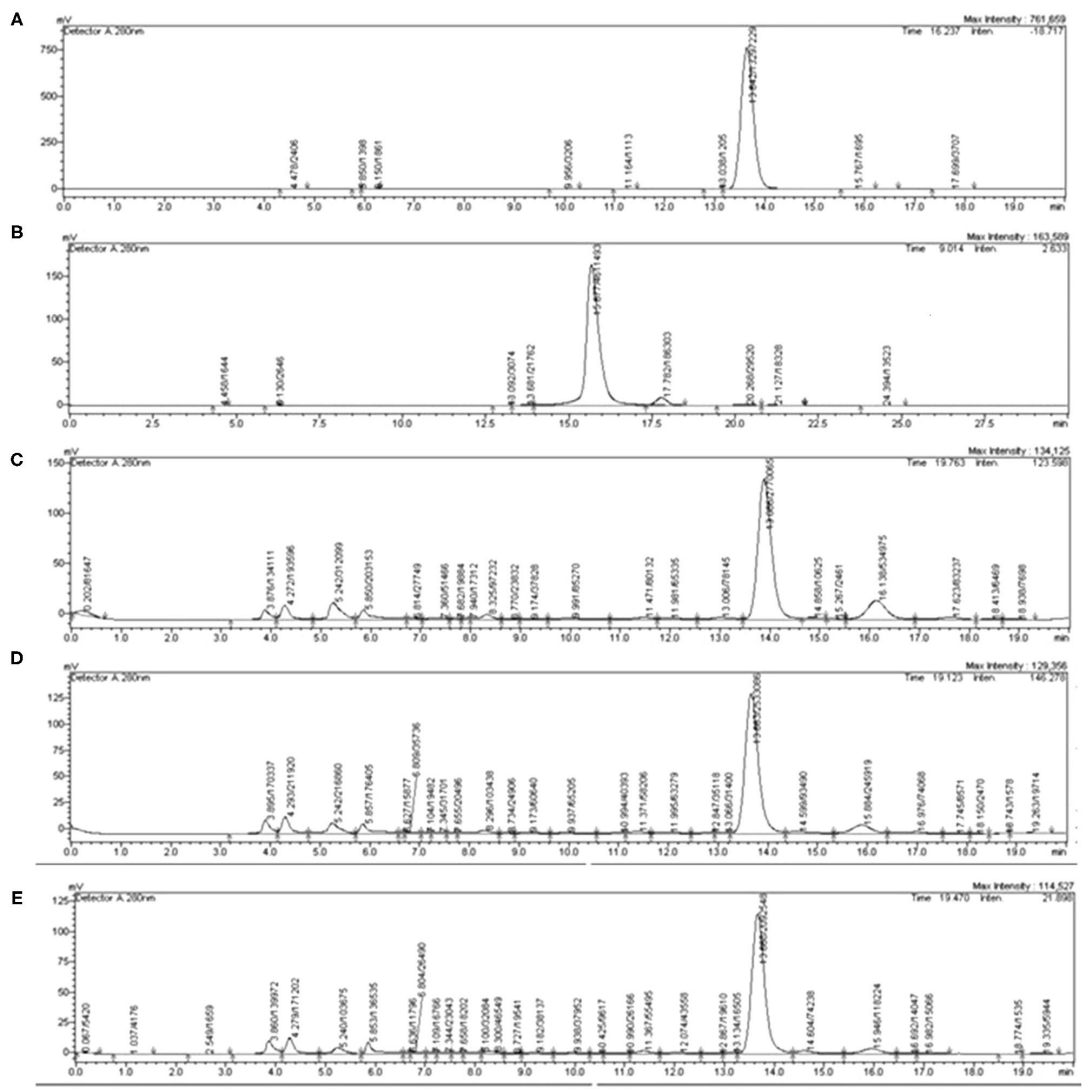
Representative high-performance liquid chromatography (HPLC) chromatograms of **(A)** caffeine, **(B)** chlorogenic acid, **(C)** light coffee (LC), **(D)** medium coffee (MC), and **(E)** dark coffee (DC). Caffeine was detected at 280 nm and chlorogenic acid was detected at 320 nm.

The typical chromatograms and the amount of caffeine and chlorogenic acid in coffee roast extracts are shown in Figure 1 and Table 3. The retention time periods of caffeine and chlorogenic acid were 13.15–13.87 min and 15.16–16.14 min, respectively. The calibration curves were *y* = 90488*x* + 3 × 10^6^ (*R*_2_ > 0.990) for caffeine and *y* = 44358*x* – 96001 (*R*_2_ > 0.998) for chlorogenic acid. The level of caffeine and chlorogenic acid tended to decrease during roasting, especially caffeine at 20°C for 5 min. However, the contents varied depending on the UAE temperature and time. The maximum caffeine content−27.65 mg/g extract—was in MC extracted at 80°C for 5 min. The highest chlorogenic acid level−16.67 mg/g extract—was in LC extracted at 80°C for 10 min.

**Table 3:** Quantitative high-performance liquid chromatography (HPLC) analysis caffeine and chlorogenic acid in coffee roast extracts.

	**Extraction temperature (^**°**^C)/extraction time (min)**	**Retention time (min)**	**Caffeine (mg/g extract)**	**Retention time (min)**	**Chlorogenic acid (mg/g extract)**	
Light coffee (LC)	20°C 5 min	13.42	19.95	15.41	15.49	
	40°C 5 min	13.77	17.68	15.90	14.08	
	80°C 5 min	13.83	24.09	15.98	16.15	
	20°C 10 min	13.82	19.12	15.98	14.14	
	40°C 10 min	13.85	19.05	16.04	13.74	
	80°C 10 min	13.84	25.68	16.03	16.66	
	20°C 20 min	13.85	19.90	16.01	14.97	
	40°C 20 min	13.81	19.05	16.07	14.49	
	80°C 20 min	13.87	19.56	16.14	14.22	
Medium coffee (MC)	20°C 5 min	13.76	17.18	15.92	14.11	
	40°C 5 min	13.33	19.28	15.34	8.96	
	80°C 5 min	13.20	27.65	15.20	8.90	
	20°C 10 min	13.17	22.74	15.17	8.96	
	40°C 10 min	13.15	22.66	15.16	9.23	
	80°C 10 min	13.29	14.26	15.37	8.87	
	20°C 20 min	13.62	6.63	15.83	5.44	
	40°C 20 min	13.75	11.48	16.00	10.38	
	80°C 20 min	13.66	16.94	15.88	7.71	
Dark coffee (DC)	20°C 5 min	13.64	14.86	15.85	5.47	
	40°C 5 min	13.63	15.72	15.85	5.32	
	80°C 5 min	13.62	13.38	15.85	4.45	
	20°C 10 min	13.64	ND	15.91	2.48	
	40°C 10 min	13.66	17.93	15.92	5.62	
	80°C 10 min	13.65	17.38	15.91	4.95	
	20°C 20 min	13.65	13.17	15.92	5.24	
	40°C 20 min	13.67	5.95	15.95	3.85	
	80°C 20 min	13.67	12.07	15.95	4.83	

The polyphenol content of coffee roast extracted at the different UAE temperatures and time periods are presented in Table 4. Interestingly, the LC and MC extracts had a higher polyphenol content than the DC extracts. However, for each coffee roast, the different extraction temperatures did not change the polyphenol content. Furthermore, except for the DC extract, the polyphenol content of the extracts tended to decrease as the extraction time increased: the content was often higher for 5-min compared with 10- and 20-min extraction time periods.

**Table 4:** Total polyphenol content of coffee roast extracts according to the extraction temperature and time.

	**Extraction temperature (^**°**^C)**	**Polyphenol [Gallic acid equivalence (GAE) mg/g extract]**		
		**5 min**	**10 min**	**20 min**
Light coffee (LC)	20	46.33 ± 2.49	41.24 ± 0.99	44.10 ± 1.56
	40	48.34 ± 10.04	42.46 ± 1.05	39.91 ± 1.47
	80	45.10 ± 1.24	45.52 ± 1.55	42.36 ± 1.35
Medium coffee (MC)	20	49.67 ± 2.03	43.42 ± 1.74	40.94 ± 1.29
	40	47.53 ± 1.52	42.79 ± 1.33	39.89 ± 0.74
	80	50.39 ± 1.92	45.80 ± 1.20	45.10 ± 1.14
Dark coffee (DC)	20	35.60 ± 1.66	34.27 ± 1.62	36.49 ± 0.78
	40	38.98 ± 1.74	39.39 ± 1.18	27.51 ± 1.06
	80	35.43 ± 1.38	34.79 ± 1.08	36.77 ± 2.68

*Data are expressed as mean ± SEM*.

Antioxidant activity at the different extraction temperatures was determined using the DPPH radical, ABTS radical, and FRAP assays (Table 5). Since the 5-min extraction time yielded the coffee roast extracts with the highest polyphenol content, these extracts were specifically chosen for the antioxidant study. From our experimentation, all coffee roast extracts were found to exhibit a great strength of the antioxidant property. From our observation, the MC extract appeared to have the most effective antioxidant activity based on DPPH and ABTS radical scavenging, whereas LC had the highest value quantified by the FRAP assay. The values obtained for DPPH and ABTS radical scavenging were similar. Regarding the DPPH and ABTS radical scavenging values, there was a variation in antioxidant activity at the different UAE temperatures, where those obtained by ABTS were lower. However, the FRAP values were similar at all UAE temperatures. It is possible that the extraction temperature had little effect on the antioxidant properties of the extracts.

**Table 5:** Antioxidant activity of coffee roasts extracts by 2,2′-diphenyl-1-picrylhydrazyl (DPPH), 2,2′-azino-bis(3-ethylbenzothiazoline-6-sulfonic acid) (ABTS), and ferric reducing antioxidant power (FRAP) assays.

	**Extraction temperature (^**°**^C)**	**Antioxidant activity**		
		**DPPH radical scavenging activity IC_**50**_ (μg/ml)**	**ABTS^**+**^ radical scavenging activity IC_**50**_ (μg/ml)**	**FRAP (μM Fe^**2+**^/g extract)**
Light coffee (LC)	20	69.41 ± 1.84	34.11 ± 1.11	186.23 ± 0.31
	40	137.35 ± 2.14	20.50 ± 1.30	189.22 ± 0.22
	80	112.8 ± 2.05	6.80 ± 1.22	189.07 ± 0.55
Medium coffee (MC)	20	51.50 ± 1.71	1.37 ± 7.04	159.85 ± 0.40
	40	52.18 ± 1.72	6.24 ± 1.14	159.29 ± 0.65
	80	51.74 ± 1.71	8.07 ± 1.24	149.59 ± 0.54
Dark coffee (DC)	20	90.42 ± 1.96	18.54 ± 1.19	156.59 ± 0.95
	40	80.47 ± 1.91	16.96 ± 1.10	152.06 ± 0.38
	80	102.77 ± 2.01	20.76 ± 1.06	156.00 ± 0.59
Trolox		4.57 ± 0.19	3.61 ± 1.02	355.28 ± 0.31

## Discussion

The results of this study, which tested the temperatures of extraction (20, 40, and 80°C) and extraction durations (5, 10, and 20 min), show that roasting conditions have a significant impact on coffee's physicochemical and biological properties. The DC extract had a lower acidity than the MC and LC extracts. Therefore, the importance of the extraction process cannot be overlooked; nevertheless, the kind of coffee roast may have a significant impact.

Coffee and its bioactive compounds are known to exert various beneficial pharmacological effects on humans (22). These biological effects might be attributed to their various micronutrients including caffeine, chlorogenic acid, trigonelline, tryptophan alkaloids, diterpenes, and other secondary metabolites. To name a few, these compounds have been scientifically revealed to protect against cardiovascular disease along with various biological properties, such as antidiabetic, neuroprotective, anticancer, antioxidant, antimicrobial effects, etc. (8, 23). The coffee brewing process is known to affect the yield, chemical composition, and biological activity of the coffee extract (24). The efficiency of the UAE in the extraction process is being lauded for, however, the integration of optimum level of UAE influence in the process of extraction is a great challenge to produce a desirable brew.

For the current study, mainly a total of three types of roasted coffee beans known as light, medium, and dark were subjected to the extraction process along with the integration of UAE at various temperatures and time periods. Following that, the physicochemical and biological characteristics of differential roasted coffee extracts were examined. Namely, the pH, °Brix, antioxidant activities, polyphenol content, and antioxidant activity. The analysis of each coffee roast showed rather consistent levels in fiber, ash, fat, protein, NFE, and energy contents. However, the moisture content of coffee beans was reduced along with the prolonged roasting process: the declining trend was observed from LC to DC. The finding is rather in complement with the existing idea of the prolonged roasting process affecting the moisture content (9). Herawati et al. (9) reported that the moisture content declined from 1.15 g/100 g on a dry basis (DB) for the light level to 0.75 g/100 g DB for the dark level. In addition, Pittia et al. (25) revealed the deteriorated moisture content during roasted light to roasted dark as 1.81 to 1.29 % (w/w). The moisture content indicates the characteristics of roasted beans; a low value indicates inadequate water content in coffee beans, and the roasted beans may be breakable and immature, whereas a high moisture content generates viscose and a hardened case that forms on the surface of coffee beans (9, 25).

Meanwhile, the change in pH and °Brix was examined as well. From our examination, the LC extract was found to be more acidic than the MC and DC extracts; however, a non-significant observable change in pH was observed when tested on varying temperatures and time periods. The trend of reducing acidity in the more roasted beans is found to be consistent with the pre-existing study (26). Our study also agrees with Rao et al. (10), who found that roasting had more of an influence on the acidity of the brew than the water extraction temperature. Whereas the cold brew coffee (extraction at room temperature) was less acidic than hot brew coffee (extraction at 100°C). However, our finding was inconsistent with these data that the acidic values were quite similar at different temperature extraction. The total soluble solids of the coffee roast extracted, measured in °Brix, did not differ between the LC, MC, and DC extracts. This observation was consistent with previous studies that reported no difference in the total solid content for different roasting processes (27). Our results indicated that the UAE extraction time and temperature did not alter the total soluble solids in the extracts.

According to the HPLC chromatogram, the caffeine content was low when the coffee beans were extracted at a low temperature (20°C, 5 min). Our results supported the study by Hečimović et al. (28) that lightly roasted coffee beans contained the highest caffeine content; the level decreased with more intense roasting. In other studies, the caffeine concentration for cold brewing was increased while it was decreased for hot brewing (100°C) (10). Another study reported that the caffeine content was stable during roasting (9). In the present study, the caffeine content varied depending on the UAE temperature and time. Chlorogenic acid was reduced with the roasting degree; the MC extract had the highest content. This content is related to potent antioxidant activity and a strong total polyphenol content in the MC extract, specifically on the extraction procedure at 80°C for 5 min. As per understanding, the roasting conditions play a crucial role in the chlorogenic acid content. The chlorogenic acid content would be diminishing along with the increased roasting intensity (26, 29). This phenomenon might be reflected in our current experimentation where the caffeine and chlorogenic acid contents might exhibit a strong correlation with the total polyphenol content, which is deeply affected by the roasting conditions.

In our initiatives to examine these potential changes, the Folin–Ciocalteu method was used to assess the total polyphenol content, and the DPPH and ABTS radical scavenging and the FRAP assays were performed to determine the antioxidant activity of the coffee roast extracts. The total polyphenol content was reduced with roasting, from LC, to MC, to DC. This result was similar to previous studies, namely that roasting decreased the polyphenol concentration (11, 27). Further observations demonstrated that the MC extract showed a strong antioxidant capacity based on DPPH and ABTS radical scavenging; the antioxidant capacity was found to decrease in the DC extract. For the FRAP assay, the LC extract had the highest antioxidant activity. However, there was variation in the activity depending on the extraction temperature. In a previous study, the highest polyphenolic content appeared in both light and medium roasting conditions (28). The highest antioxidant activity was found in the medium-roasted coffee by the ABTS radical scavenging, whereas the maximum activity was found in the light-roasted coffee by the DPPH radical scavenging (30). Vignoli et al. (12) suggested that distinct activities depend on the method used to measure antioxidant activity and the assessment of antioxidant activity should be evaluated by more than one method. They found that antioxidant activity based on the ABTS radical scavenging decreased as the roasting degree increased, whereas the activity remained stable with the FRAP assay. The diminished antioxidant activity may be associated with the loss of phenolic compounds during the roasting process (12).

Taken all together, our findings shed light on the importance of temperature and extraction processing time point selection by pinpointing the levels of change in caffeine and polyphenol contents. From our observation, the highest levels of caffeine and polyphenol were recorded at the extraction time point of 5 min with a temperature of 80°C. These variables were found to be dropped or illegally degraded when being processed for a long time period as observed at 10 and 20 min of MC. These observation and results implied that the extraction process under influence of the UAE had a significant effect on the extractive level of the biologically active compound. Therefore, a rather short and higher temperature condition is much preferable for the production of products with great strength in biological potential.

## Conclusion

The present study revealed that roasting conditions play an important role in the physicochemical and biological characteristics of coffee. Along the way, the importance of the extraction process cannot be omitted as well; however, it could be in great reliance on the type of coffee roast. There was variation in the pH, antioxidant activity, total phenolic content, and caffeine and chlorogenic acid contents at differential UAE extraction temperatures and time points. The LC and MC extracts exhibited strong antioxidant activity and a high total polyphenol content and maximum caffeine and chlorogenic acid contents. The measured antioxidant activities depended on the method used to assess antioxidant activity. The caffeine and chlorogenic acid contents were associated with the total polyphenol content and depend on the roasting and extraction conditions. Our findings represent comprehensive reports on the physicochemical and biological characteristics of roasted coffee extracts under the influence of UAE, which is deemed useful for the production of a specific brew by not compromising its biological potential.

## Data Availability Statement

The original contributions presented in the study are included in the article/supplementary material, further inquiries can be directed to the corresponding author/s.

## Author Contributions

AD contributed to designing the study. AD and SS participated in the acquisition, analysis, interpretation of data, and drafting the manuscript. SS, B-HG, and PP revised the manuscript for important intellectual content. All authors revised the manuscript critically for important intellectual content and approved the final version.

## Conflict of Interest

The authors declare that the research was conducted in the absence of any commercial or financial relationships that could be construed as a potential conflict of interest.

## Publisher's Note

All claims expressed in this article are solely those of the authors and do not necessarily represent those of their affiliated organizations, or those of the publisher, the editors and the reviewers. Any product that may be evaluated in this article, or claim that may be made by its manufacturer, is not guaranteed or endorsed by the publisher.
